# Human Lung Fibroblasts Exhibit Induced Inflammation Memory *via* Increased *IL6* Gene Expression and Release

**DOI:** 10.3389/fimmu.2022.921728

**Published:** 2022-07-22

**Authors:** Jennifer Maries Go Yap, Takashi Ueda, Yoshihiro Kanemitsu, Norihisa Takeda, Kensuke Fukumitsu, Satoshi Fukuda, Takehiro Uemura, Tomoko Tajiri, Hirotsugu Ohkubo, Ken Maeno, Yutaka Ito, Testsuya Oguri, Shinya Ugawa, Akio Niimi

**Affiliations:** ^1^ Department of Respiratory Medicine, Allergy and Clinical Immunology, Nagoya City University Graduate School of Medical Sciences, Aichi, Japan; ^2^ Department of Anatomy and Neuroscience, Nagoya City University Graduate School of Medical Sciences, Aichi, Japan

**Keywords:** inflammatory memory, fibroblasts, trained immunity, SARS-CoV-2, Poly (I:C), interleukin-6

## Abstract

Fibroblasts of different origins are known to possess stromal memory after inflammatory episodes. However, there are no studies exploring human lung fibroblast memory which may predict a subsequent inflammatory response in chronic respiratory diseases and COVID-19. MRC-5 and HF19 human lung fibroblast cell lines were treated using different primary and secondary stimulus combinations: TNFα–WD–TNFα, Poly (I:C)–WD–TNFα, TNFα–WD–Poly (I:C), or LPS–WD–TNFα with a 24-h rest period (withdrawal period; WD) between the two 24-h stimulations. TLR3 and NF-κB inhibitors were used to determine pathways involved. The effect of SARS-Cov-2 spike protein to inflammatory response of lung fibroblasts was also investigated. mRNA expressions of genes and IL6 release were measured using qRT-PCR and ELISA, respectively. Statistical significance was determined by using one- or two-way ANOVA, followed by Bonferroni’s *post hoc* analysis for comparison of multiple groups. Preexposure with Poly (I:C) significantly increased TNFα-induced *IL6* gene expression and IL6 release in both cell lines, while it affected neither gene expressions of *IL1B*, *IL2*, *IL8*, and *MMP8* nor fibrosis-related genes: *ACTA2*, *COL1A1*, *POSTN*, and *TGFB1*. Inhibition of TLR3 or NF-κB during primary stimulation significantly downregulated IL6 release. Simultaneous treatment of MRC-5 cells with SARS-CoV-2 spike protein further increased TNFα-induced IL6 release; however, preexposure to Poly (I:C) did not affect it. Human lung fibroblasts are capable of retaining inflammatory memory and showed an augmented response upon secondary exposure. These results may contribute to the possibility of training human lung fibroblasts to respond suitably on inflammatory episodes after viral infection.

## Introduction

Immune memory has been initially described in acquired immune systems (1), but increasing evidence indicates that innate immunological memory also exists in immune cells such as dendritic cells and macrophages (2). Moreover, this type of memory can also occur in stromal cells such as endothelial cells, epithelial cells, and fibroblasts, although it is limited compared to professional innate immune cells. Crowley et al. (2018) defined inflammatory memory as modification in the capacity of stromal cells to respond to an inflammatory stimulus such as tumor necrosis factor alpha (TNFα) or an exogenous stimulus such as viruses or endotoxins (3). There are different studies investigating inflammatory memory using endothelial cells challenged with polyinosinic polycytidylic acid [Poly (I:C)] and rechallenged with lipopolysaccharide (LPS) (4) and also using epithelial basal cells challenged with interleukin 4 (IL4)/interleukin 13 (IL13) (5). In contrast, fibroblast inflammatory memory has been reported in a limited origin of fibroblasts. Human gingival fibroblasts were shown to maintain production of interleukin 6 (IL6) and interleukin 8 (IL8) by secondary LPS treatment, even when pretreated with LPS (6). Also, diseased tendon fibroblasts treated with interleukin 1 beta (IL1β) sustained the *IL6* and *IL8* mRNA expressions (7). Fibroblast-like synoviocytes (FLS) derived from patients with rheumatoid arthritis (RA) displayed enhanced production of certain cytokines and chemokines when preexposed to TNFα and subsequent interferon (IFN) stimulation (8). In addition, a study done on FLS from both inflamed and non-inflamed joints also exhibited inflammatory memory with augmented IL6 release upon TNFα restimulation (9). However, there are no reports exploring the possibility that lung fibroblasts may retain an inflammatory memory.

Lung fibroblasts are known to play an important role in chronic respiratory diseases in which majority of conditions involve inflammation (10). They possess the ability to produce inflammatory cytokines, chemokines, and antimicrobial peptides (11, 12). Several viruses that infect the lungs, such as avian influenza virus, severe acute respiratory syndrome coronavirus (SARS-Cov), and respiratory syncytial virus, may produce high levels of proinflammatory cytokines (13). The mechanism of coronavirus disease 2019 (COVID-19) and other respiratory diseases starts from an excessive release of cytokines and chemokines including IL6, TNFα, IL8, transforming growth factor beta (TGFβ), and matrix metalloprotease 9 (MMP9) (14–16) which triggers a cascade of inflammatory responses and cytokine storm, followed by pneumonia, severe damage to the airways, pulmonary edema, and eventually acute respiratory distress syndrome (ARDS), making the disease fatal (17–19). TNFα is an important cytokine involved in immunity and known to act as an amplifier of inflammation (17) while IL6 was identified as the most correlated cytokine to severe and critical COVID-19 conditions (18). During lung inflammation followed by ARDS, the activation of the IL6-mediated positive feedback loop of NF-κB signaling in non-immune cells such as fibroblasts known as IL6 amplifier plays a critical role in inducing cytokine storm as observed in severe COVID-19 patients (19). Moreover, a previous study demonstrated that Poly (I:C) with SARS-Cov-2 spike protein mimicked COVID-19-induced ARDS and cytokine storm syndrome in murine models (20).

A recent study reported that duration of disease is an important determinant for lung fibrosis post ARDS (21), suggesting that a previous inflammatory event is maintained in locally accessible cell types as memory. The possibility that inflammatory memory is stored in stromal cells present during infection particularly lung fibroblasts may shed light to training these cells to respond appropriately in an inflammatory state to prevent exacerbated responses as seen during cytokine storm.

We hypothesized that human lung fibroblasts are capable of response based on inflammatory memory which may be associated with lung fibrosis induced by cytokine storm after SARS-Cov-2 infection. The aim of this study is to explore the presence of inflammatory memory in lung fibroblasts and investigate the underlying genes involved in causing such responses upon primary and secondary stimulus.

## Materials and Methods

### Cell Culture and Treatment of Cells

**Figure 1:**
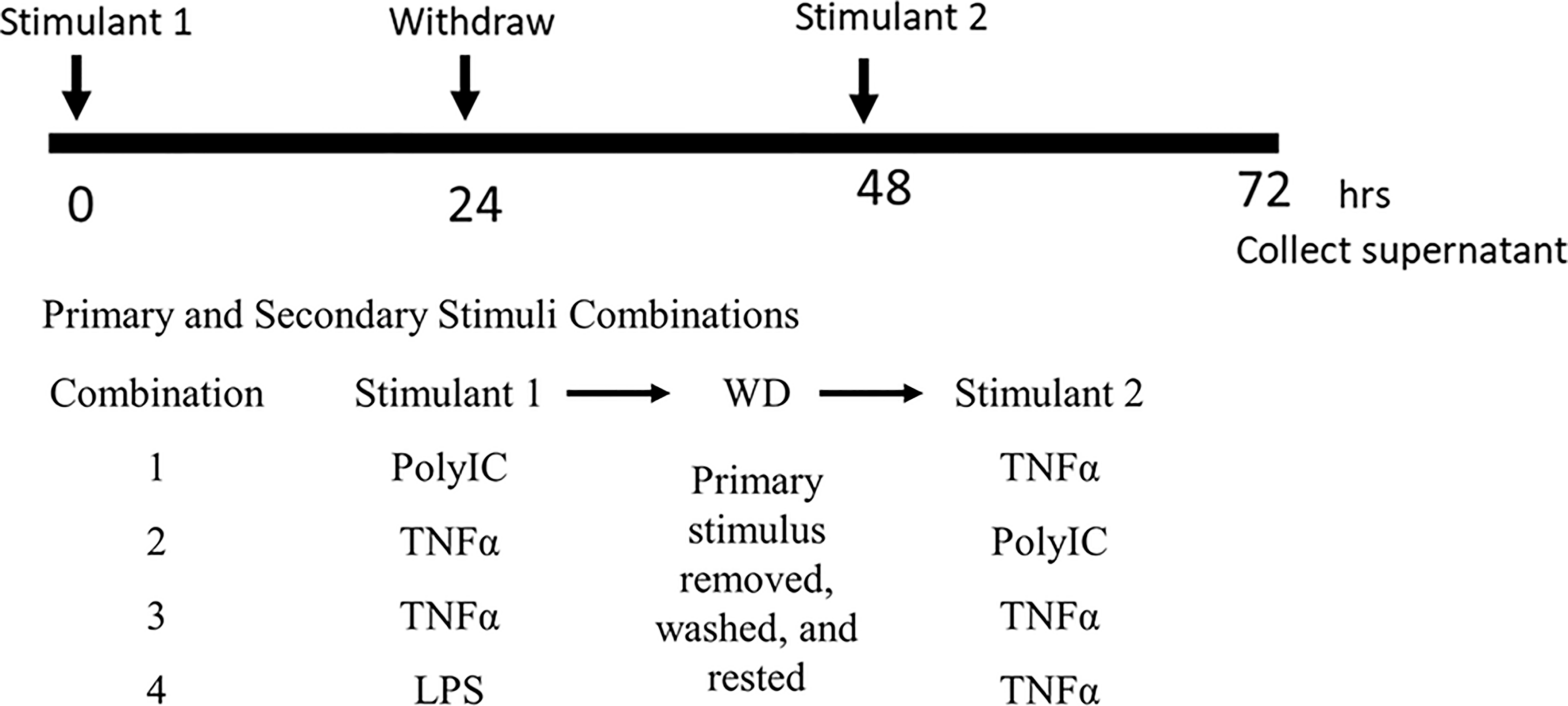
Experimental treatment procedure. Different combinations of stimulants were utilized to investigate the presence of inflammatory memory in human lung fibroblasts. Withdrawal method means that stimulus 1 was removed, and cells were washed. TLR3 and NF-κB inhibitors were administered together with Stimulus 1.

MRC-5 and HF19 cells were provided by RIKEN BRC through the National Bio-Resource Project of the MEXT/AMED, Japan (RCB021 and RCB0210). MRC-5 (Medical Research Council cell strain 5) is a diploid cell culture line composed of fibroblasts, originally developed from the lung tissue of a 14-week-old aborted Caucasian male fetus. MRC-5 cells themselves are known to reach senescence in around 45 population doublings (PDL). The HF19 or human fetal lung fibroblast-like cell line is established from a 14-week-old female fetus and are known to reach senescence at 16 PDL. Both cell lines were cultured in RITC80-7 medium [IFP 0160, IFP (Research Institute for the Functional Peptides), Yamagata, Japan] supplemented with 10% FCS (04-001-1A, Biological Industries, Beit HaEmek, Israel), penicillin (P7794, Sigma-Aldrich, St. Louis, MO, USA), and streptomycin (S9137, St. Louis, MO, USA) at 37°C with 5% CO_2_. Cells were seeded in a six-well plate with 1% FCS RITC80-7 medium for quantitative RT-PCR (qRT-PCR) and Western blot analysis, and in a 96-well plate for ELISA with serum free RITC80-7 medium. Cells were treated with different stimuli such as Poly (I:C) (0, 1, or 10 µg/ml) (4287, Tocris Bioscience, Bristol, UK), and TNFα (0, 1, 5, or 10 ng/ml) (300-01A, PeproTech, Rocky Hill, NJ, USA) in a manner shown in **Figure 1**. Briefly, cells were initially incubated with first stimuli for 24 h then washed with fresh medium twice, had a 24-h rest period (withdrawal; WD), and were exposed to second stimuli for another 24 h. The following combinations were examined in this study: TNFα–WD–TNFα (TNF–WD–TNF), Poly (I:C)–WD–TNFα (PolyIC–WD–TNF), TNFα–WD–Poly (I:C) (TNF–WD–PolyIC), or LPS–WD–TNFα (LPS–WD–TNF). These cells were also subjected to different conditions using inhibitors such as TLR3/dsRNA complex inhibitor (TLR3i, 10 µM) (614310, EMD Millipore, Darmstadt, Germany) and BAY11-0782 (1.5 µM) (AG-CR1-0013-M010, AdipoGen Life Sciences, San Diego, CA, USA) together with the initial stimulation for different experiments. Cells were incubated for a total of 72 h before harvest. Poly (I:C) is a synthetic dsRNA analogue that is used commonly for models of viral infections *in vivo*. TNFα was used to assess the effect of the presence of an inflammatory mediator during this condition. In some experiments, SARS-CoV-2 spike protein (3 µg/ml) (Z03481, GenScript, Piscataway, NJ, USA) was used as a stimulus to check its effect on PolyIC–WD–TNF in MRC-5 cells.

### RNA Isolation and cDNA Synthesis

MRC-5 cells were treated in the same manner as shown in **Figure 1**. The cells were then harvested at 0 h (immediately), 6 h, 12 h, and 24 h after secondary TNFα treatment. Isolation of total RNA was done using ISOGEN reagent according to the manufacturer’s protocol (Fujifilm Wako, Osaka, Japan). SuperScript IV VILO Master Mix with ezDNase kit was used to synthesize cDNA following the manufacturer’s protocol (11766050, Invitrogen by Thermo Fisher Scientific, Waltham, MA, USA). One thousand five hundred nanograms of total RNA was used to synthesize cDNA.

### Quantitative RT-PCR of Target Genes

Quantitative RT-PCR (qRT-PCR) of target genes were performed utilizing the 7900HT Fast real-time PCR system (Applied Biosystems, Thermo Fisher Scientific, USA). We established specific primers as described in **Supplementary Table 1**. Relative gene expressions were calculated using the 2^-ΔΔCt^ method after normalization with *ACTB* (β-actin) values.

### Cell Viability and Enzyme-Linked Immunosorbent Assay

Cells were plated in 96-well plates (2 × 10^4^ cells/well) and preexposed with Poly (I:C) for 24 h followed by withdrawal for 24 h and then treated with or without TNFα for 24 h at 37°C. For control groups, a vehicle solution (DMSO) with or without preexposure to Poly (I:C) and TNFα was applied to cells. Cell Counting Kit 8 (CCK8) was used to check the number of live cells per well. Relative release of IL6 was measured using the ELISA kit according to the manufacturer’s protocol (KE00139, Proteintech, Chicago, IL, USA). Briefly, 100 μl of standards or samples (2× dilution) from each well was incubated for 2 h at room temperature. Washing was done every after incubation. Then the samples were incubated for 1 h with human IL6 detecting antibody and then with HRP conjugate for 40 min. Incubation with TMB substrate was done for 18 min. After adding the stop solution, the absorbance at 450 nm with a wavelength correction at 540 nm was measured with a SpectraMax 340 plate reader (Molecular Devices, San Jose, CA, USA). The values were compared against a standard curve that was generated using known concentrations of IL6 to calculate concentration in the samples (in pg/mL) and normalized with the respective CCK8 value.

### Western Blot Analysis

MRC-5 cells were treated in the same manner as shown in **Figure 1**. The cells were then harvested at 0 min (immediately), 15 min, 30 min, and 60 min after secondary TNFα treatment. Total protein samples (20 μg) were resolved by electrophoresis using SDS-polyacrylamide (10%) gel (EHR-T10L, ATTO, Tokyo, Japan) and were transblotted onto Immobilon-P membrane (IPVH00010, Merck Millipore, Dublin, Ireland) using semi-dry transfer (Trans-Blot SD Cell, Bio-Rad, USA). Five percent of skim milk–Tris-buffered saline with 0.3% Tween-20 (TBST) was used for blocking. The membranes were incubated with primary antibodies such as anti-NF-κB p65 (D14E12) [8242, Cell Signaling Technology (CST), Tokyo, Japan], anti-STAT3 (79D7) (4904, CST, Japan), anti-phospho-NF-κB p65 (Ser 536) (93H1) (3033, CST, Japan), and anti-phospho-STAT3 (Tyr 705) (D3A7) (9145, CST, Japan) followed by secondary anti-rabbit IgG HRP-conjugated antibody (7074S, CST, Japan). The bands were detected using chemiluminescence kit (ECL detection system; GE Healthcare, Chicago, IL, USA) and were quantified using ImageJ software.

###  Statistical Analysis

GraphPad Prism 5 statistical software was used to analyze data that were represented as means ± SEM. The results were treated using one- or two-way ANOVA, followed by Bonferroni’s *post-hoc* analysis for comparison of multiple groups to check for statistical significance. P < 0.05 was considered statistically significant. Experiments were done in triplicates or more for reproducibility.

## Results

### Cross-Stimulation With Poly (I:C) and TNFα Induced *IL6* Gene Expression in Human Lung Fibroblasts

**Figure 2:**
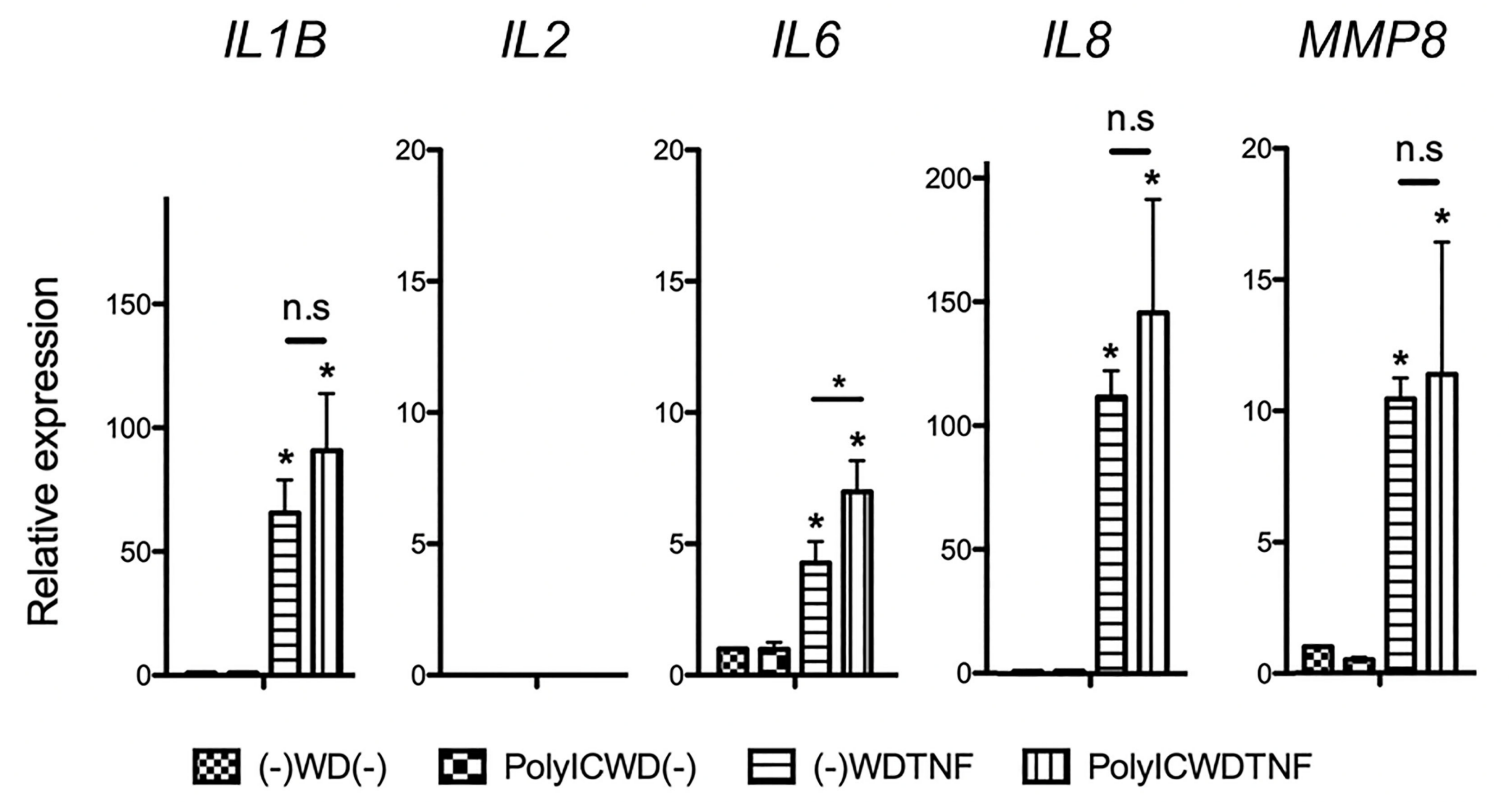
Gene expressions after PolyIC–WD–TNF in MRC-5 cells. MRC-5 cells were initially treated with Poly (I:C) (10 µg/ml) for 24 h and were then washed and rested for 24 h before being secondarily treated with TNFα (10 ng/ml) for another 24 h. Poly (I:C) alone [PolyIC–WD-(-)] did not increase any of the gene expressions investigated, while TNFα alone [(-)-WD–TNF] variably induced the gene expressions of *IL1B*, *IL6*, *IL8*, and *MMP8*, but not *IL2*. Preexposure to Poly (I:C) significantly increased TNFα-induced *IL6* expressions (P < 0.05) but not *IL1B*, *IL8*, and *MMP8*. N = 4. Values with * were defined significant when compared to (-)-WD-(-) while *above bar were significant between two variables; n.s means not significant.

To determine the presence of inflammatory memory in human lung fibroblasts, we utilized human lung fibroblast cell lines, MRC-5 cells, and HF19 cells. Our experimental design is similar to the study of Crowley et al. (2017) also using fibroblasts (9). The withdrawal method (stimulus was removed, and cells were washed and had a 24-h rest period between two stimulations) was used to check the effect of reexposing the cells to the same or different stimuli (**Figure 1**). Both cell lines were initially subjected to Poly (I:C) (10 μg/ml) then removed and was secondarily treated with TNFα (10 ng/ml) after a 24-h withdrawal. In MRC-5 cells, Poly (I:C) alone [PolyIC–WD-(-)] did not increase any of the gene expressions investigated, whereas TNFα alone [(-)-WD–TNF] significantly increased the gene expressions of *IL1B*, *IL6*, *IL8*, and *MMP8* (P < 0.05). Moreover, preexposure of MRC-5 cells to Poly (I:C) significantly enhanced TNFα-induced *IL6* gene expressions (P < 0.05), but not *IL1B*, *IL8*, and *MMP8*. No expression of the *IL2* gene was observed in all combinations (**Figure 2**). Time-course experiments showed that induction of *IL6* and *IL8* gene expressions was not found at 0 h, peaked at 6 h, and declined toward 24 h while *MMP8* gene expression gradually increased toward 24 h after a second treatment with TNFα only [(-)-WD–TNF] (**Supplementary Figure 1**). Pretreatment with Poly (I:C) evoked early gene inductions of *IL6* and *IL8* at 0 h (immediately after secondary treatment with TNFα) (Poly–WD–TNF) (**Supplementary Figure 1A**). In addition, *IL6* gene expression was increased at 6 h and sustained at 12 and 24 h (**Supplementary Figure 1B**). In contrast, *IL8* gene expression was increased at 6 h and gradually decreased toward 24 h (**Supplementary Figure 1C**) as *MMP8* gene expression was gradually increased toward 24 h after pretreatment with Poly (I:C) and secondary treatment with TNFα (Poly–WD–TNF) (**Supplementary Figure 1D**). These results showed that *IL6* exhibited a distinct pattern of gene expression to TNFα in human lung fibroblasts when cells were preincubated with Poly(I:C).

**Figure 3:**
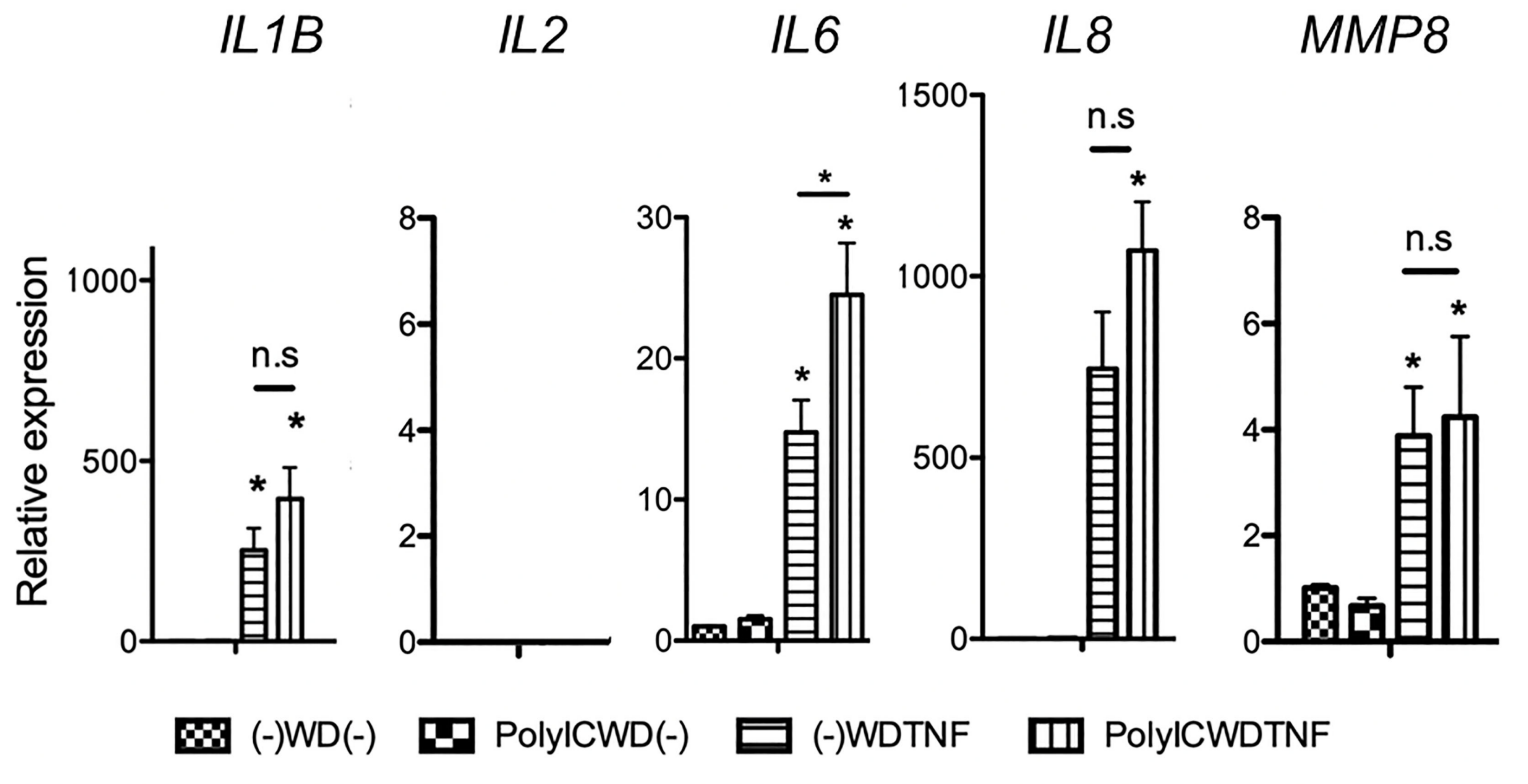
Gene expressions after PolyIC–WD–TNF in HF19 cells. HF19 cells were initially treated with Poly (I:C) for 24 h and were then washed and rested for 24 h before secondarily treated with TNFα for another 24 h. Preexposure of HF19 cells to Poly (I:C) significantly increased TNFα-induced *IL6* expressions (P < 0.05) but not *IL1B*, *IL8*, and *MMP8*. N = 3. Values with * were defined significant when compared to (-)-WD-(-) while *above bar were significant between two variables; n.s means not significant.

HF19 cells also exhibited similar responses to MRC-5 cells. Briefly, TNFα alone [(-)-WD–TNF] induced expressions of all genes examined (P < 0.05) except for *IL2* and preexposure with Poly (I:C) (10 μg/ml) showed upregulated *IL6* gene expressions (P < 0.05) but not of *IL1B*, *IL8*, and *MMP8* (**Figure 3**).

**Figure 4:**
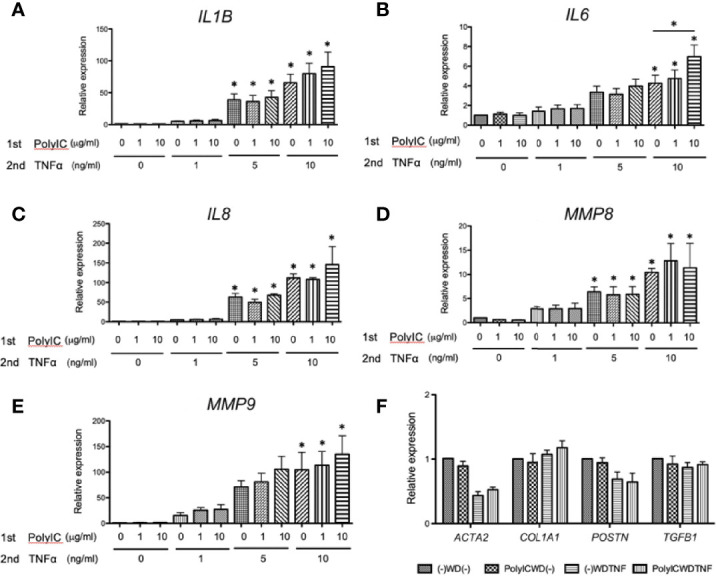
Gene expressions after PolyIC–WD–TNF at different concentrations of Poly (I:C) and TNFα. MRC-5 cells were initially treated with Poly (I:C) for 24 h and were then washed and rested for 24 h before being secondarily treated with TNFα for another 24 h. **(A–E)** Effect of different concentrations of Poly (I:C) (0, 1, 10 µg/ml) and TNFα (0, 1, 5, 10 ng/ml) on gene expressions of *IL1B*, *IL6*, *IL8*, *MMP8*, and *MMP9*. Secondary treatment to TNFα dose-dependently induced these gene expressions. Preexposure to Poly (I:C) did not affect TNFα-induced *IL1B*, *IL8*, *MMP8*, and *MMP9* gene expressions [PolyIC (0 µg/ml)–WD–TNF (10 ng/ml) vs. PolyIC (10 µg/ml)–WD–TNF (10 ng/ml)]. **(F)** Preexposure to Poly (I:C) (10 µg/ml) and secondary treatment of MRC-5 cells with TNFα (10 ng/mL) did not affect the fibrosis-related genes; *ACTA2*, *COL1A1*, *POSTN*, and *TGFB1*. N = 4. Values with * were defined significant when compared to (-)-WD-(-) while *above bar were significant between two variables.

It is possible that the genes related to inflammation may exhibit similar gene upregulations to *IL6* at a different concentration of Poly (I:C) and TNFα. Therefore, we further confirmed if different concentrations of Poly (I:C) (0, 1, 10 μg/ml) and TNFα (0, 1, 5, 10 ng/ml) could elucidate a dose-dependent upregulation. The Poly (I:C) concentration at 10 μg/ml and TNFα concentration at 10 ng/ml produced a significantly upregulated *IL6* gene expression, indicating optimal conditions for our experiments (P < 0.05), although this experimental condition still failed to increase the gene expressions of *IL1B*, *IL8*, *MMP8*, and *MMP9* despite using different concentrations (**Figures 4A–E**). However, we can observe that these gene expressions responded dose-dependently to secondary treatment of TNFα (5 and 10 ng/ml) (P < 0.05), suggesting that the increase of TNFα during infection may synergistically aggravate production of other cytokines (**Figures 4A–E**). We also examined the effect of this stimulus combination on expression of fibrosis-related genes, but gene expressions of *ACTA2* [alpha smooth muscle actin (α-SMA)], *COL1A1* (collagen type 1 alpha 1), *POSTN* (periostin), and *TGFB1* (TGFβ1) were not increased (**Figure 4F**).

### Preexposure of Human Lung Fibroblasts to Poly (I:C) Significantly Increased TNFα-Induced IL6 Release

**Figure 5:**
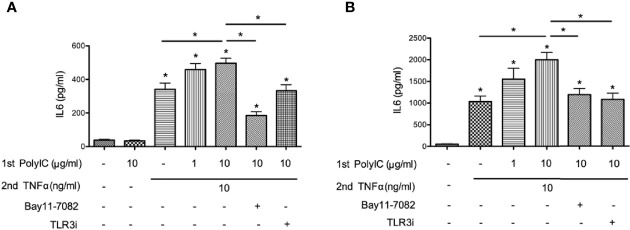
IL6 release after PolyIC–WD–TNF in **(A)** MRC-5 cells and **(B)** HF-19 cells and the effect of TLR3 and NF-κB inhibitors on its release. Preexposure to Poly (I:C) significantly increased TNFα-induced IL6 release in both MRC-5 (P < 0.005) and HF-19 (P < 0.005) cells. Both inhibitors significantly decreased IL6 release in MRC-5 cells (P < 0.01 and P < 0.0001 for TLR3i and Bay-11-7082, respectively) and HF-19 cells (P < 0.01 and P < 0.05 for TLR3i and Bay-11-7082, respectively). N = 6. Values with * were defined significant when compared to (-)-WD-(-) while *above bar were significant between two variables.

To determine the effect of Poly (I:C) cross-stimulation with TNFα on release of inflammatory mediators, we performed IL6 release assay using the ELISA kit. MRC-5 cells were treated first with or without Poly (I:C) (10 μg/ml) followed by a 24-h withdrawal and further treated with or without TNFα (10 ng/ml). Poly (I:C) alone did not increase IL6 release (**Figure 5A**). However, preexposure of MRC-5 cells to Poly (I:C) (10 μg/ml) significantly increased TNFα-induced IL6 release (P < 0.005) as compared to TNFα alone-induced IL6 release (**Figure 5A**). Poly (I:C) 1 μg/ml tended to increase TNFα-induced IL6 release but was not significant. The increased response was also observed in another human lung fibroblast cell line, HF19 cells (**Figure 5B**).

### NF-κB and TLR3 Inhibitors Significantly Downregulated IL6 Release

MRC-5 cells were reported to produce IFNβ upon stimulation with Poly (I:C) and express its receptor, TLR3, on the cell surface (22). The activation of TLR3 by Poly (I:C) leads to activation of the transcription factors interferon regulatory factor 3 (IRF3) and NF-κB *via* the adapter molecule TRIF (23). In addition, NF-κB activation is a major regulator of TNFα-stimulated IL6 expression (24). To check whether these pathways govern the preexposure of human lung fibroblasts to Poly (I:C)-increased TNFα-induced IL6, we used inhibitors of TLR3 (TLR3i) and NF-κB (Bay-11-7082). MRC-5 and HF-19 cells were treated in the same manner as in **Figure 1** with or without TLR3i and Bay11-7082 together with the first stimulus. In both lung fibroblast cell lines, TLR3i and Bay11-7082 significantly downregulated TNFα-induced IL6 release after preexposure to Poly (I:C) (P < 0.01 and P < 0.0001 in MRC-5 cells, respectively) (**Figure 5A**) (P < 0.01 and P < 0.05 in HF19 cells, respectively) (**Figure 5B**). These results signify that these pathways may be responsible for the aggravated immune responses.

To further check the involvement of NF-κB, we examined NF-κB p65 phosphorylated at Serine-536 (p-NF-κB p65) after secondary TNFα treatment through Western blot analysis. Results showed that NF-κB p65 was activated at 15 min and sustained activation until 60 min after the second TNFα treatment [(-)-WD–TNF and Poly–WD–TNF]. However, there was no significant difference between (-)-WD–TNF and Poly–WD–TNF (**Supplementary Figure 2**). The result implied that the activation of NF-κB p65 in response to TNFα was maintained in lung fibroblasts pretreated with Poly(I:C) during the examined early time points.

Signal transducer and activator of transcription 3 (STAT3) was identified as an IL6-activated transcription factor and activated by phosphorylation at Tyr-705 in the transactivation domain (25). STAT3 is known for the induction of IL6 (26). We performed Western blot analysis to investigate if pretreatment with Poly (I:C) can activate STAT3. Our results showed that phospho-STAT3 at Tyr-705 tended to increase at all time points analyzed when pretreated with Poly (I:C) (Poly–WD–TNF), although there was no significant difference between (-)-WD–TNF and PolyIC–WD–TNF (**Supplementary Figure 2**).

### Preexposure to TNFα Did Not Induce *IL6* Gene Expression, but Significantly Increased IL6 Release

**Figure 6:**
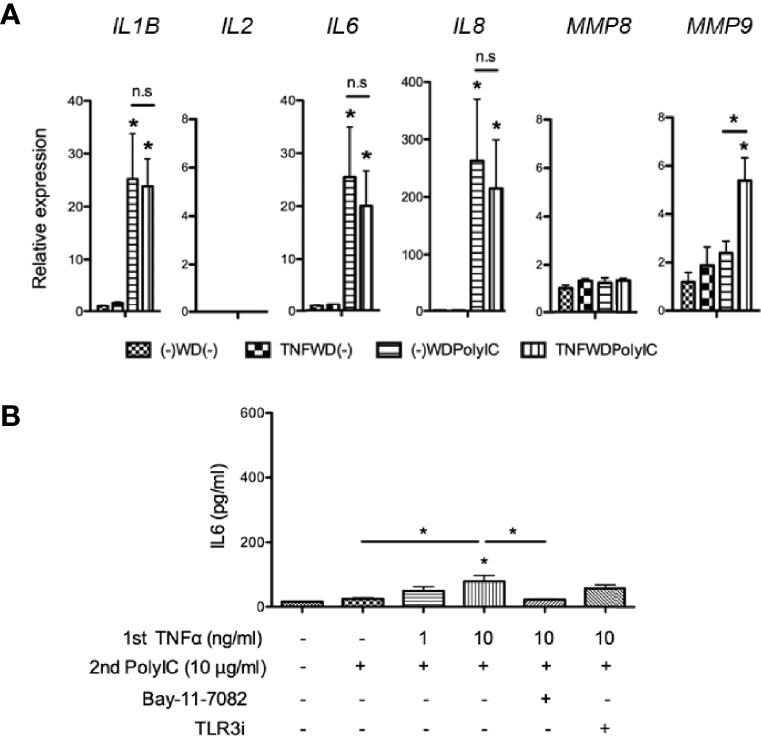
Stimulation of MRC-5 cells using TNF–WD–PolyIC. **(A)** Gene expressions of *IL1B*, *IL2*, *IL6*, *IL8*, *MMP8*, and *MMP9*. Exposure to Poly (I:C) increased the gene expressions of *IL1B*, *IL6*, and *IL8* (P < 0.005), but not those of *IL2*, *MMP8*, and *MMP9* [(-)-WD-(-) vs. (-)-WD–PolyIC]. Pretreatment with TNFα did not enhance the cytokine gene expressions. However, *MMP9* expression was significantly increased (P < 0.05) [(-)-WD–PolyIC vs. TNF–WD–PolyIC]. N = 4. **(B)** IL6 release was significantly increased upon preexposure to TNFα (10 ng/ml) and secondarily treated with Poly (I:C), However, low levels of IL6 were detected (< 100 pg/ml). N = 4. Values with * were defined significant when compared to (-)-WD-(-) while *above bar were significant between two variables.

In order to check if the effect of preexposure to Poly(I:C) was specific, we reversed the stimulation and used TNFα first to treat MRC-5 cells and secondarily treated them with Poly (I:C). Poly(I:C) significantly increased the gene expressions of *IL1B*, *IL6*, and *IL8* [(-)-WD-(-) vs. (-)-WD–PolyIC] (P < 0.05), but preexposure to TNFα did not change the gene expressions [(-)-WD–PolyIC vs. TNF–WD–PolyIC] (**Figure 6A**). In contrast, *MMP9* gene expression was upregulated when preincubated with TNFα as compared to Poly (I:C) alone-treated cells [(-)-WD–PolyIC vs. TNF–WD–PolyIC] (**Figure 6A**). As demonstrated in our previous study (27), *MMP9* gene expression was positively regulated by TNFα treatment in MRC-5 cells and may synergistically increase its expression together with Poly (I:C).

We then performed IL6 release assay; however, TNFα cross-stimulation with Poly (I:C) released low levels of IL6 with a maximum at less than 100 pg/ml [TNF (10)–WD–PolyIC] (**Figure 6B**). Although low levels of IL6 were detected, preexposure of MRC-5 cells to TNFα significantly upregulated IL6 release. Simultaneous treatment of Bay-11-7082 with primary TNFα significantly inhibited the increased IL6 release, suggesting that the NF-κB pathway could be involved in this process.

We also examined gene expressions and IL6 release using another combination, TNF–WD–TNF, to check the specificity of the response. Even using this combination, preexposure to TNFα did not affect the gene expressions examined after secondary stimulation with TNFα (**Supplementary Figure 3**). Moreover, TNFα alone [(-)-WD–TNF] significantly increased IL6 release, but pretreatment with of TNFα did not further increase TNFα-induced IL6 release (TNF–WD–TNF) (**Supplementary Figure 4**), which may indicate that Poly (I:C) represents a specific mediator to augment TNFα-induced IL6 release in human lung fibroblasts.

### Secondary Treatment of MRC-5 Cells With SARS-CoV-2 Spike Protein Further Increased TNFα-Induced IL6 Release

**Figure 7:**
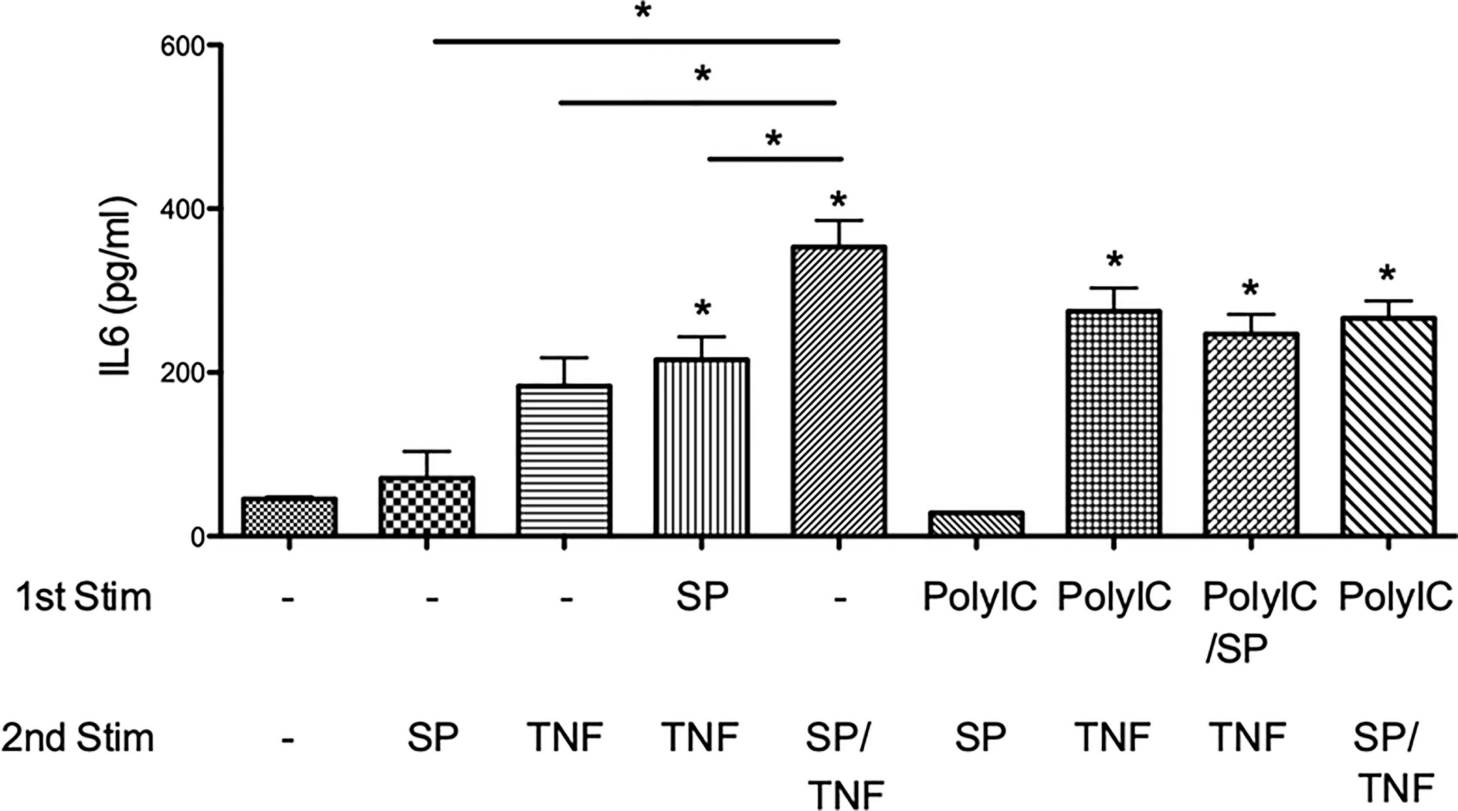
Effect of SARS-CoV-2 spike protein (SP) on IL6 release. SP alone did not increase IL6 release [(-)-WD–SP]. Preexposure to SP did not affect TNFα-induced IL6 release [(-)-WD–TNF vs. SP–WD–TNF], but co-stimulation of SP with TNFα significantly increased IL6 release [(-)-WD–TNF vs. (-)-WD–SP/TNF] (P < 0.05). Preexposure to Poly (I:C) did not affect the TNFα-induced IL6 release. N = 6. Values with * were defined significant when compared to (-)-WD-(-) while *above bar were significant between two variables.

We further examined this phenomenon with the presence of the synthetic SARS-CoV-2 spike protein (SP) in order to determine its effect on TNFα-induced IL6 release. MRC-5 cells were treated in the same manner (**Figure 1**) with or without SP. **Figure 7** shows that the secondary treatment with SP alone [(-)-WD–SP] did not induce significant IL6 release. Also, preexposure with SP and secondary treatment with TNFα (SP–WD–TNF) failed to increase it; however, the second treatment of TNFα with SP [(-)-WD–SP/TNF] showed further IL6 release as compared to [(-)-WD–TNF]. This result shows the synergistic effect of TNFα and SP in increasing IL6 release. Furthermore, preexposure to Poly (I:C) did not influence the secondary treatment of SP [PolyIC–WD–SP vs. (-)-WD–SP] and also the TNFα-induced IL6 release after pretreatment with Poly (I:C) (PolyIC/SP–WD–TNF and PolyIC–WD–SP/TNF) (**Figure 7**).

## Discussion

The present study demonstrated for the first time that human lung fibroblasts could retain inflammatory memory. This inflammatory memory induces an augmented IL6 response rather than a negative “tolerized” response when treated with Poly (I:C) and TNFα. It was only specific to certain combinations such as PolyIC–WD–TNF and TNF–WD–PolyIC. Neither was it observed in the TNF–WD–TNF combination, nor was a different ligand (LPS–WD–TNF: data not shown) used. The PolyIC–WD–TNF combination produced a greater effect on IL6 release than the TNF–WD–PolyIC combination. The response of lung fibroblasts to an inflammatory stimulus was hypothesized to be influenced by the pattern of its exposure (6). In addition, this inflammatory memory was specific to the IL6 gene and not observed in other genes investigated (*IL1B*, *IL8*, and *MMP8*). Various types of fibroblasts (synovial and dermal fibroblasts) showed different memory-like responses to pro-inflammatory cytokine (9), suggesting that preexposure to Poly (I:C) triggered a memory-like response to increase TNFα-induced IL6 release in human lung fibroblasts. Altogether, the observed human lung fibroblast memory in this study displays a distinctive character as it is specific to stimulus, cell, and gene.

The intracellular mechanism of stromal memory in human lung fibroblasts is still unknown. Poly (I:C) is a synthetic analog of viral dsRNA (28). It is recognized by three intracellular receptors: an endosome-located TLR3 and two cytoplasmic receptors, retinoic acid–inducible gene I (RIG-I) and melanoma differentiation–associated protein-5 (MDA-5) (29). TLR3 was detected on the cell surface of MRC-5 cells (22) and human lung fibroblasts (30). Both Poly (I:C) and TNFα can activate various signaling pathways (NF-κB, and MAPK signaling) that lead to the production of proinflammatory cytokines (31, 32). These signaling pathways could be involved in the augmented IL6 response. As a result, we utilized inhibitors for TLR3 and NF-κB and found that both inhibitors could reduce the increased TNFα-induced IL6 release when each inhibitor was pretreated together with Poly (I:C) (**Figure 5**). Thus, preexposure to Poly (I:C) could trigger human lung fibroblasts to enhance TNFα-induced IL-6 release *via* TLR3 and NF-κB pathways.

Our study raises the question of what mechanism governs the specificity of the inflammatory memory observed. NF-κB is important for the activation of the *IL6* gene (33) and plays a role in signaling of IL-6 production in lung fibroblasts cocultured with mast cells (34). Recently, a study by DeFelice et al. (2021) reported on the molecular mechanism that shows appropriate stimulus- and dose-dependent dynamics *via* NF-κB oscillation. Non-oscillatory NF-κB activation in IkBa-deficient bone marrow-derived macrophages (BMDMs) provoked robust gene induction in 58% of total genes as compared with wild-type BMDMs showing oscillatory NF-κB activation (35). A previous study reported that nuclear translocation of NF-κB/p65 was already detected in MRC-5 human lung fibroblasts after exposure to TNFα (2 ng/mL) for 1 h (36). We then examined the phosphorylation of NF-κB p65 during the early phase (0–60 min) after secondary treatment with TNFα. Our result showed that NF-κB p65 was promptly phosphorylated by secondary treatment with TNFα. The activation was maintained, but not increased in the cells pretreated with Poly(I:C). We recommend investigating not only the rapid phosphorylation but also the slow and persistent activation of NF-κB pathways throughout all the time points [after Poly(I:C), withdrawal, or TNFα]. Thus, we still believe that NF-κB oscillation and signaling dynamics may govern the capability of human lung fibroblasts to retain inflammatory memory. With the development of new optogenetic tools and single-cell analysis, NF-κB signaling dynamics throughout all stages should be investigated in human lung fibroblasts.

In addition to NF-κB pathways, STAT3 signaling may be involved in this process, as the positive feedback loop of IL-6 signaling (the IL-6 amplifier) was activated by the simultaneous stimulation of NF-κB and STAT3 in mouse embryonic fibroblasts (37, 38). Poly (I:C) may have indirectly activated STAT3 signaling and switched on the IL6 amplifier, leading to the further activation of IL6 release While we observed background Tyr-705 phosphorylated STAT3 (p-STAT3) in untreated cells [(-)-WD-(-)], the activation of STAT3 in MRC-5 lung fibroblasts treated with PolyIC–WD–TNF showed a trend which was slightly higher than that of cells treated with (-)-WD–TNF at all the time points examined. Since STAT3 activation was reported to be affected by cell density and serum starvation in cell cultures (39, 40), optimizing experimental conditions for a total of 72 h of incubation of cells is recommended for further studies.

**Figure 8:**
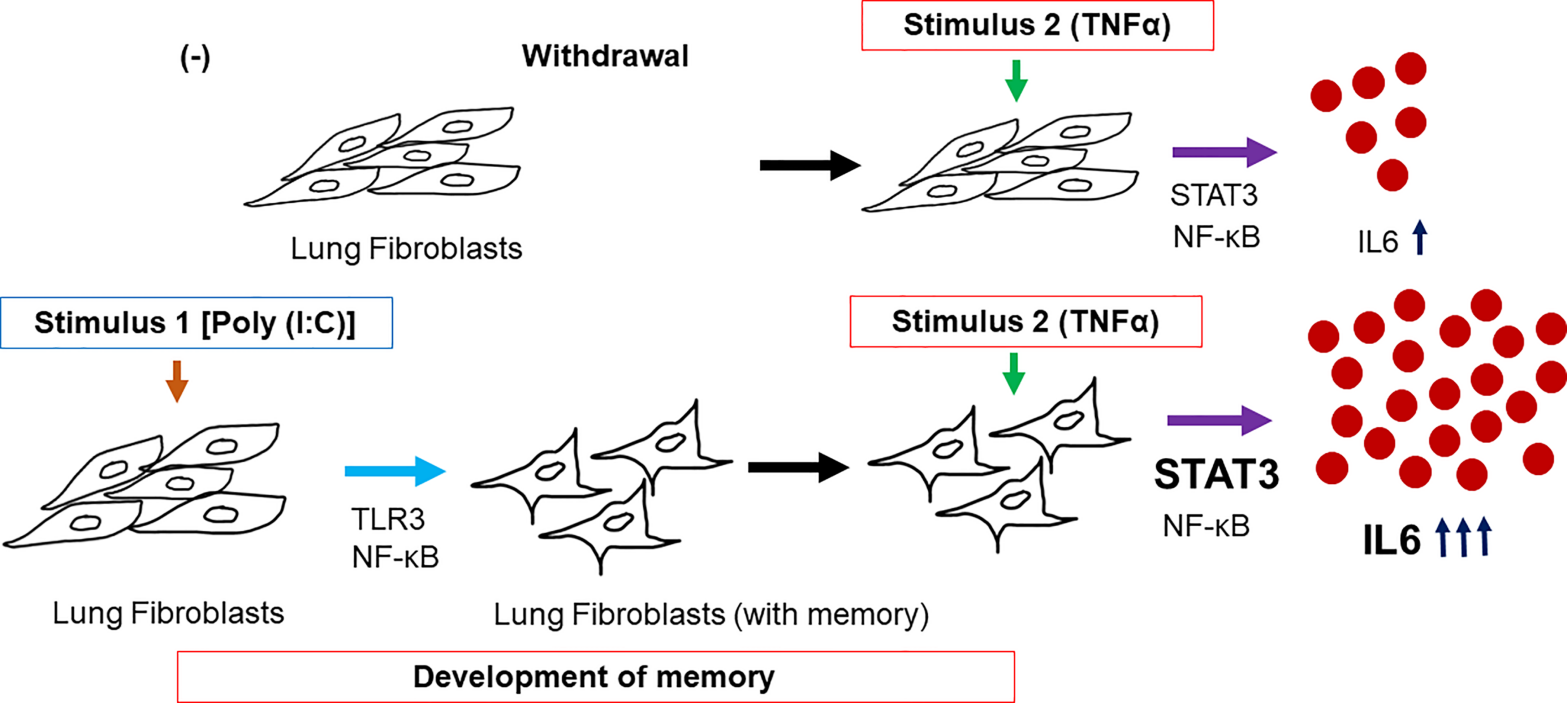
Model summarizing the possible mechanism in the development of inflammatory memory in human lung fibroblast as exhibited by increased IL6 response. Pretreatment with Poly (I:C)-activated TLR3 and NF-κB pathways and responses were stored by the lung fibroblasts. Upon secondary treatment of the cells with memory, STAT3 and NF-κB were simultaneously activated further increasing IL6 response.

A model summarizing the possible mechanism of the reported inflammatory memory in human lung fibroblasts is shown in **Figure 8**. Pretreatment with Poly (I:C) activates TLR3 and NF-κB in human lung fibroblasts which may have been stored as a memory. Upon second treatment with TNFα, the fibroblasts with memory then simultaneously activates both NF-κB and STAT3 increasing IL6 response which may initiate autocrine response and further activating STAT3. This phenomenon may be responsible for the cytokine storm observed in COVID-19 and other chronic respiratory diseases (19).

The present study also showed that the synthetic SARS-CoV-2 spike protein (SP) in combination with TNFα increased the release of IL6 levels. It suggests that SP can synergize with other cytokines to induce cytokine storms. However, pretreatment with Poly (I:C) or SP did not affect TNFα-induced IL6 release. Thus, we could not find evidence for the presence of inflammatory memory using these experimental conditions. Further studies are needed to investigate this inflammatory response. On the other hand, preexposure to Poly (I:C) may switch TNFα-induced cytokine release to fibrotic response in the presence of SP.

The human lung fibroblast memory should be investigated further using different markers of inflammatory response and longer time intervals within the treatments. Using different TLR ligands and other stimulations may further deepen the knowledge on fibroblast memory. Additional investigations on the use of synthetic SARS-CoV-2 spike protein variants are needed in order to determine if human lung fibroblast memory is involved in the occurrence of long-COVID.

These results contribute to the development of new therapeutic approaches that may specifically train human lung fibroblasts to inhibit inflammatory responses and enhance pathogen defenses and immune tolerance. These may be beneficial to patients suffering from inflammatory-induced diseases such as SARS-CoV-2 infection and other chronic respiratory diseases.

## Data Availability Statement

The raw data supporting the conclusions of this article will be made available by the authors, without undue reservation.

## Author Contributions

JY and TUed performed the experiments, analyzed the data, and wrote the manuscript. NT, KF, SF, TUem, TT, HO, KM, YI, and TO discussed and supervised the study. YK contributed to the design of the work and drafted and revised the manuscript. SU and AN supervised and approved the final manuscript.

## Conflict of Interest

The following authors received research grants and personal fees outside the submitted work: YK received research grants from Novartis Pharma, MSD, Sanofi, and personal fees from GSK, Novartis Pharma, AstraZeneca, Sanofi, and Kyorin. KF received research grants from Novartis Pharma and GSK. SF received personal fees from AstraZeneca and Eli Lilly. HO received a research grant from Boehringer Ingelheim. KM received personal fees from Pfizer and Chugai Pharmaceutical. TO reports personal fees from AstraZeneca, Eli Lilly Japan, Taiho Pharmaceutical, Pfizer, Chugai Pharmaceutical, MSD, Daiichi Sankyo, and Asahi Kasei Pharma, as well as research grants and personal fees from Kyowa Hakko Kirin, Boehringer Ingelheim, Ono Pharmaceutical, and Novartis. AN reports personal fees from Astellas, AstraZeneca, Kyorin, GSK, MSD, Shionogi, Bayer, Sanofi, Taiho, and Boehringer Ingelheim, and research grants from Astellas, Kyorin, Boehringer Ingelheim, Novartis, MSD, Daiichi Sankyo, Taiho, Teijin, Ono, Takeda, and Sanofi Pharmaceutical.

The remaining authors declare that the research was conducted in the absence of any commercial or financial relationships that could be construed as a potential conflict of interest.

## Publisher’s Note

All claims expressed in this article are solely those of the authors and do not necessarily represent those of their affiliated organizations, or those of the publisher, the editors and the reviewers. Any product that may be evaluated in this article, or claim that may be made by its manufacturer, is not guaranteed or endorsed by the publisher.
