# Supplementary material for: Human Lung Fibroblasts Exhibit Induced Inflammation Memory via Increased IL6 Gene Expression and Release
Source: Front Immunol. 2022 Jul 22;13:921728. doi: 10.3389/fimmu.2022.921728 (PMC9356221; doi:10.3389/fimmu.2022.921728)
Supplement: Supplementary file 1 [file DataSheet_1.docx]

Supplementary Material

# Supplementary Table 1. List of Primer Sequences used for quantitative RT-PCR in the study.

| Gene (GenBank#) | Forward sequence | Reverse sequence | bp size |
| --- | --- | --- | --- |
| Homo sapiens *COL1A1*: NM_000088.3 | AGCTTTGTGGACCTCCGGCT  133-152 | GCAGGTGATTGGTGGGATGTCT  225-246 | 114 |
| Homo sapiens *POSTN*: NM_001135934.1 | TACTGGAAACCATCGGAGGCA  1461-1481 | CGGAATATGTGAATCGCACCGT 1563-1584 | 124 |
| Homo sapiens *MMP9*: NM_004994.2 | CTTTGGACACGCACGACGTCT  1992-2012 | TCAGGGCACTGCAGGATGTCAT 2115-2136 | 145 |
| Homo sapiens  *ACTA2*: NM_001613.3 | CATTGCCGACCGAATGCAGAA 1100-1120 | CCACCGATCCAGACAGAGTAT  1184-1204 | 105 |
| Homo sapiens  *ACTB*: NM_001101.3 | TGGCACCCAGCACAATGAAGATCA 1043-1066 | CTGCTTGCTGATCCACATCTGCT 1142-1164 | 122 |
| Homo sapiens *MMP8*: NM_002424.3 | TGGACCCAATGGAATCCTTGC 621-641 | GGCCAAATTCATGAGCAGCAACA 738-760 | 140 |
| Homo sapiens *TGFB1*:  NM_000660.6 | CACGTGGAGCTGTACCAGAAAT 1338-1359 | TCAACCACTGCCGCACAACT  1445-1464 | 127 |
| Homo sapiens *IL1B*:  NM_000576.3 | GAGCAACAAGTGGTGTTCTCCA 544-565 | AACACGCAGGACAGGTACAGAT 632-653 | 110 |
| Homo sapiens *IL2*:  NM_000586.4 | AGACCCAGGGACTTAATCAGCA 586-605 | CAATGGTTGCTGTCTCATCAGCA 666-688 | 103 |
| Homo sapiens *IL6*:  NM_000600.5 | CAGAGCTGTGCAGATGAGTACA 483-504 | TCGTCAGCAGGCTGGCATTTGT 574-595 | 113 |
| Homo sapiens *IL8*:  NM_000584.4 | TCTGTCTGGACCCCAAGGAAAACT 317-340 | GCTTGAAGTTTCACTGGCATCTTCA 424-448 | 132 |

# Supplementary Figures


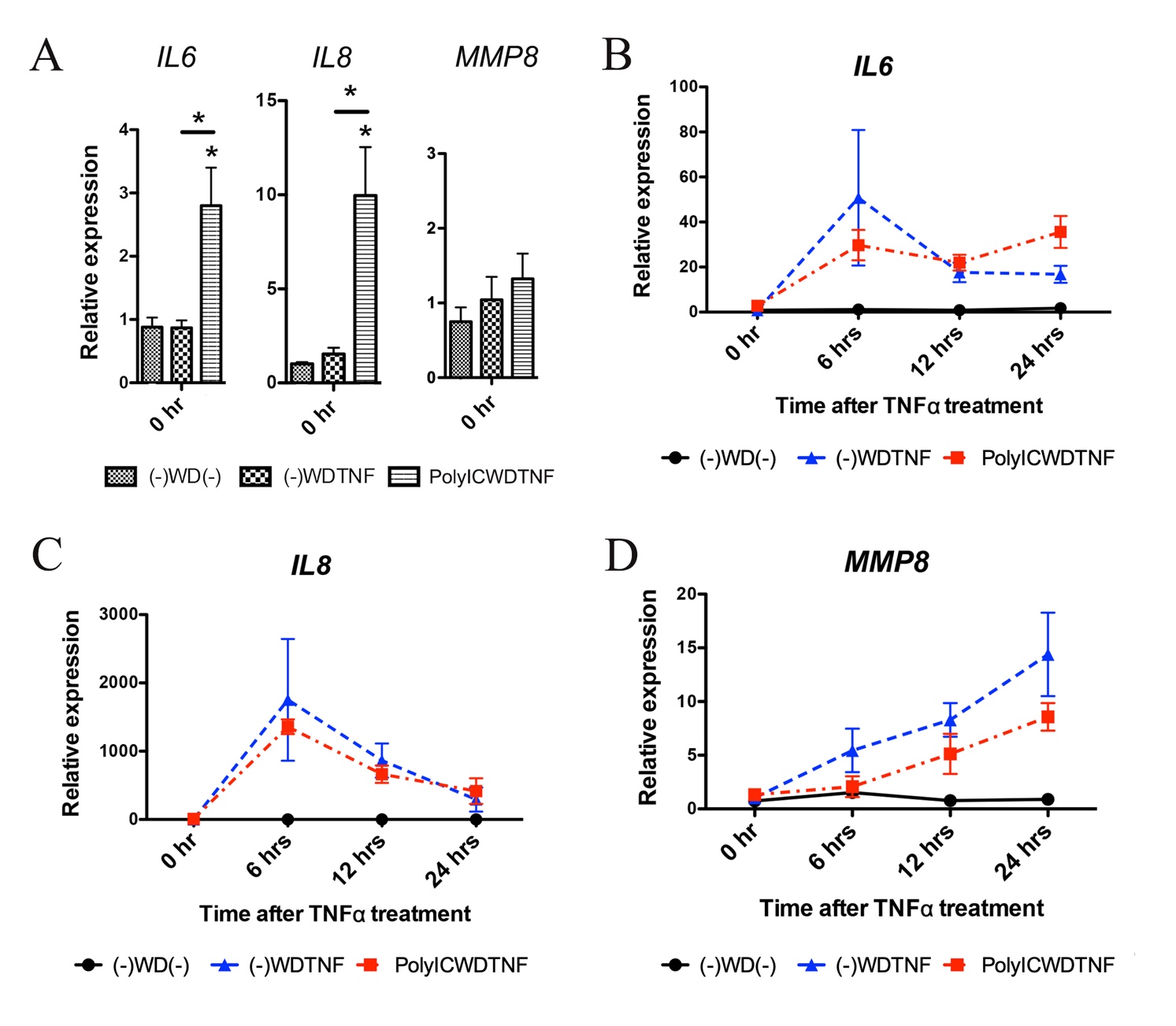


**Supplementary Figure 1.** Time Course of relative *IL6, IL8* and *MMP8* gene expressions. MRC-5 cells were harvested at 0 hr (immediately), 6 hrs, 12 hrs, and 24 hrs after secondary TNFα treatment. (A) Poly (I:C) pretreatment evoked significant inductions of *IL6* and *IL8* genes at 0 hr after TNFα treatment (PolyICWDTNF). (B-D) In cells treated with TNFα only [(-)WDTNF represented by blue dashed lines], induction of *IL6* and *IL8* gene expressions peaked at 6 h and declined towards 24 hrs while *MMP8* gene expression gradually increased towards 24 hrs. In cells stimulated with PolyIC-WD-TNF (PolyICWDTNF represented by red dash-dotted lines), *IL6* gene expression was increased at 6 hrs, and sustained at 12 hrs and 24 hrs. In contrast, *IL8* gene expression was increased at 6 hrs and gradually decreased towards 24 hrs. *MMP8* gene expression was gradually increased towards 24 hrs. * P < 0.05, N=3


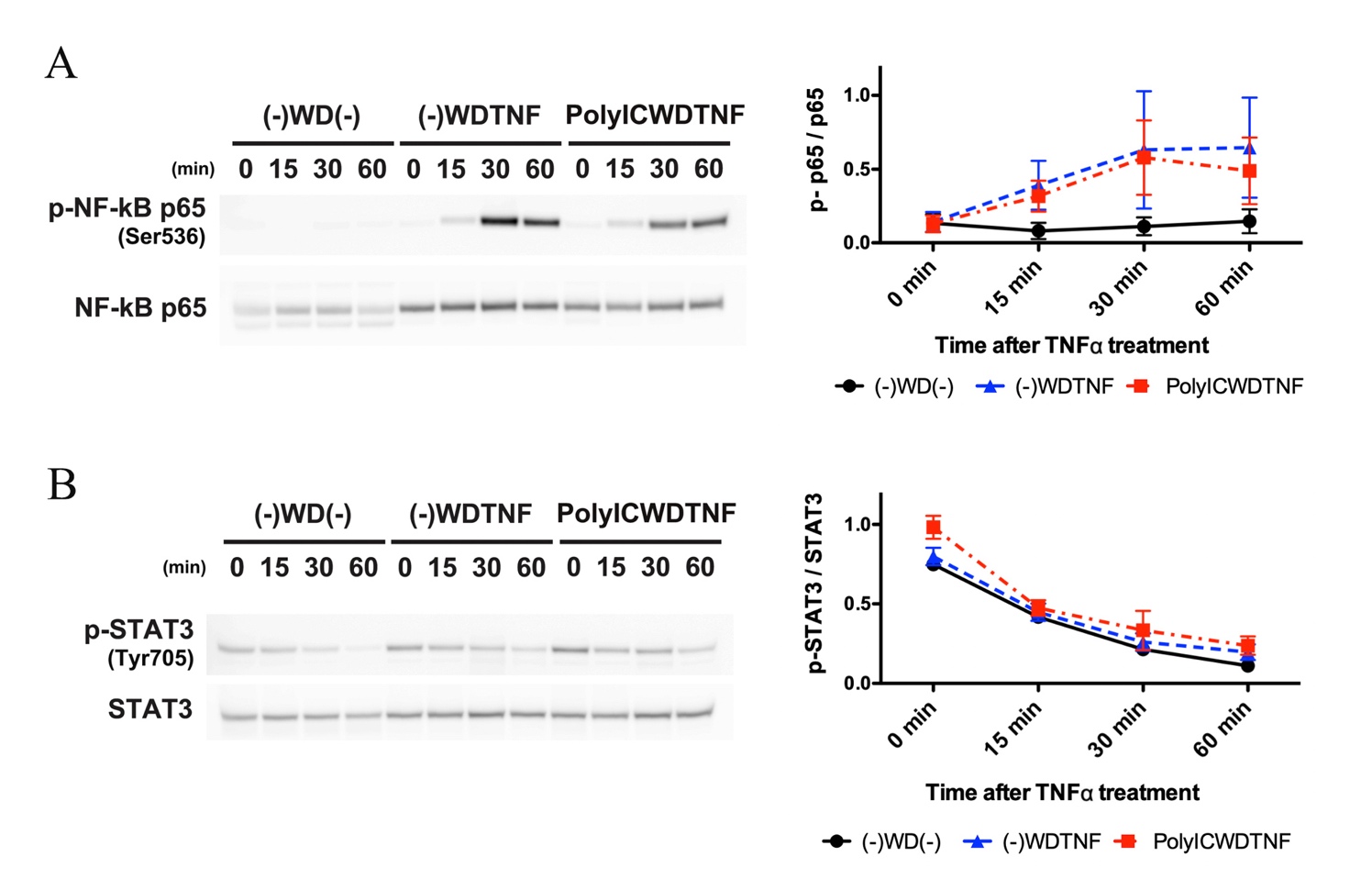


**Supplementary Figure 2.** Western blot analysis of (A) p-NF-κB p65 (Ser-536), NF-κB p65 and (B) p-STAT3 (Tyr-705), and STAT3. MRC-5 cells were harvested at 0 min (immediately), 15 min, 30 min and 60 min after secondary TNFα treatment. (A) Activation of NF-κB p65 (Phospho-NF-κB p65) was observed at 15 min and sustained until 60 min after TNFα treatment [(-)WDTNF (blue dashed lines) and PolyICWDTNF (red dash-dotted lines)]. There was no significant difference between (-)-WD-TNF and Poly-WD-TNF. (B) Phospho-STAT3 (p-STAT3) was detected immediately (0 h) and tended to increase when pretreated with Poly (I:C) (PolyICWDTNF represented by red dash-dotted lines), however, it was not significant. N=3


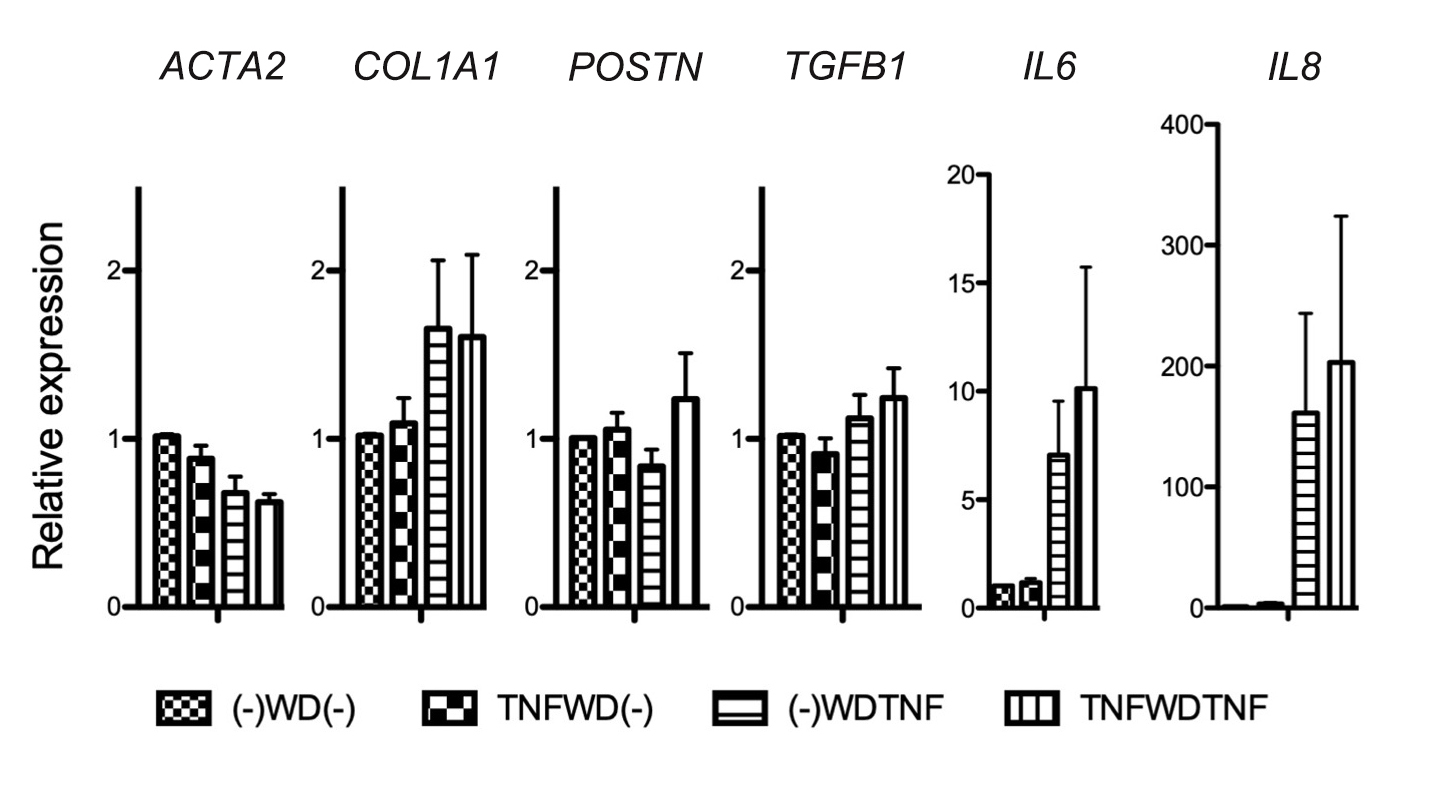


**Supplementary Figure 3.** Gene expressions after TNF-α-WD-TNF-α (TNFWDTNF) in MRC-5 cells. Pretreatment and restimulation with TNF-α neither affect gene expressions of *ACTA2, COL1A1, POSTN* and *TGFB1* nor significantly increased *IL6* and *IL8* gene expressions. N=4


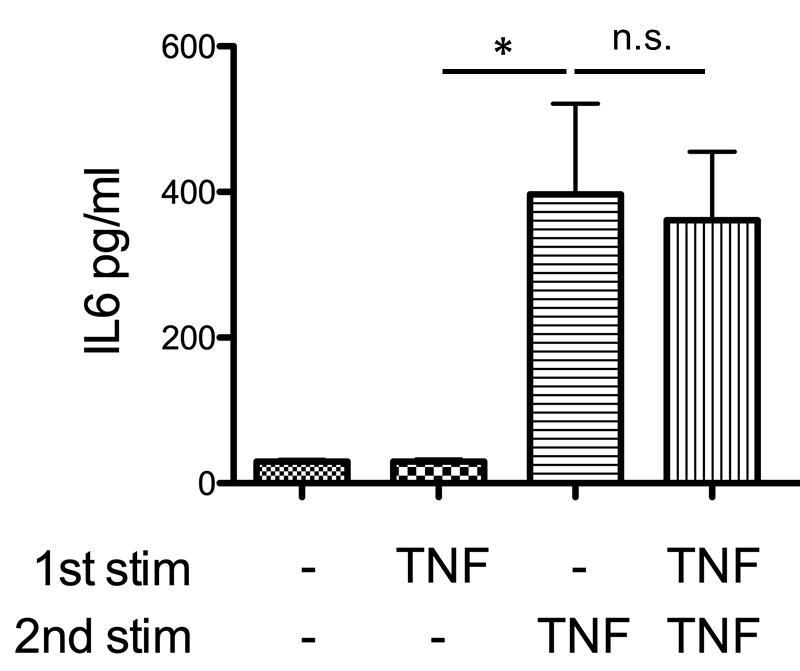


**Supplementary Figure 4.** IL6 release in MRC-5 cells stimulated with TNFα-WD-TNFα. Secondary treatment of MRC-5 cells with TNFα significantly increased IL6 release (P < 0.05), but pretreatment with TNFα did not affect the TNFα-induced IL6 release. N= 3
